# Optimization of a DNA Nicking Assay to Evaluate *Oenocarpus bataua* and *Camellia sinensis* Antioxidant Capacity

**DOI:** 10.3390/ijms151018023

**Published:** 2014-10-09

**Authors:** Louis-Jérôme Leba, Christel Brunschwig, Mona Saout, Karine Martial, Emmanuelle Vulcain, Didier Bereau, Jean-Charles Robinson

**Affiliations:** Université des Antilles et de la Guyane, UMR QUALITROP, campus universitaire de Troubiran, P.O. Box 792, 97337 Cayenne Cedex, French Guiana, France; E-Mails: ljleba@pasteur-cayenne.fr (L.-J.L.); christel.brunschwig@gmail.com (C.B.); mona_saout@hotmail.com (M.S.); karine.martial@guyane.univ-ag.fr (K.M.); ema_vulcain@yahoo.fr (E.V.); didier.bereau@guyane.univ-ag.fr (D.B.)

**Keywords:** *pUC18*, antioxidant, prooxidant, DNA nicking assay, Fenton, Amazonian palm, *Oenocarpus bataua*, *Camellia sinensis*

## Abstract

This study was aimed at assessing the DNA damage protective activity of different types of extracts (aqueous, methanolic and acetonic) using an *in vitro* DNA nicking assay. Several parameters were optimized using the pUC18 plasmid, especially FeSO_4_, EDTA, solvent concentrations and incubation time. Special attention has been paid to removing the protective and damaging effect of the solvent and FeSO_4_ respectively, as well as to identifying the relevant positive and negative controls. For each solvent, the optimal conditions were determined: (i) for aqueous extracts, 0.33 mM of FeSO_4_ and 0.62 mM of EDTA were incubated for 20 min at 37 °C; (ii) for acetone extracts, 1.16% solvent were incubated for 15 min at 37 °C with 1.3 mM of FeSO_4_ and 2.5 mM of EDTA and (iii) for methanol extracts, 0.16% solvent, were incubated for 1.5 h at 37 °C with 0.33 mM of FeSO_4_ and 0.62 mM of EDTA. Using the optimized conditions, the DNA damage protective activity of aqueous, methanolic and acetonic extracts of an Amazonian palm berry (*Oenocarpus bataua*) and green tea (*Camellia sinensis*) was assessed. Aqueous and acetonic *Oenocarpus bataua* extracts were protective against DNA damage, whereas aqueous, methanolic and acetonic extracts of *Camellia sinensis* extracts induced DNA damage.

## 1. Introduction

Reactive nitrogen species (RNS) and Reactive oxygen species (ROS), such as nitric oxide (•NO), peroxynitrite (ONOO^−^), hydrogen peroxide (H_2_O_2_), superoxide (O_2_•^−^.) and hydroxyl radical (•OH), are implicated in oxidative stress in cells, DNA damage [1], cancer [2,3], cells aging [4] and neurodegenerative disease like Alzheimer’s and Parkinson’s [5,6]. During the past decades, the oxidative stress implication in numerous diseases led to considerable efforts in identifying beverages, vegetables, fruits and plants with high antioxidant properties [7,8,9]. Antioxidant compounds can inhibit the oxidation chain at different stages: either by radical-chain breaking (direct radical quenching/scavenging), or by chelating metals that act as catalyst [10]. Currently, the antioxidant capacity is evaluated via several *in vitro* chemical assays, which measure either the oxygen radical absorbance capacity (ORAC) (peroxide radical), the 2,2-diphenylpicrylhydrazyl (DPPH) free radical-scavenging capacity and the ferric-reducing ability of plasma (FRAP). These assays are interesting because of their complementarities and the fact that they imply a variety of parameters such as different mechanisms, substrates and radicals [11,12,13]. If *in vitro* chemical assays are good tools to study antioxidant capacity of natural products, *in vitro* DNA nicking assays are more biologically relevant and they enable a fast screening of potential *in vivo* antioxidant substances. DNA nicking assay has been created because damage to the genome is central to the development of disease such as degenerative diseases and cancers [6,14,15]. Among the various existing *in vitro* DNA nicking assays, the DNA nicking assay based on the Fenton reaction mimics the *in vivo* biological situation, with the production of hydroxyl free radicals from endogenous entities like intracellular iron. The Fenton reaction was described more than 100 years ago [16]. During this reaction, H_2_O_2_ is cleaved in •OH by electron transfer from iron according to the reaction: Fe^2+^ + H_2_O_2_ → Fe^3+^ + •OH + ^−^OH. In the DNA nicking assay due to •OH formation during the reaction, which is a highly reactive and strong oxidizing species, the initial supercoiled configuration of plasmid DNA changes from supercoiled to open circular and nicked linear forms that present altered electrophoretic mobility properties on gel [17]. Nevertheless, depending on the reaction mixture composition DNA degradation is not only induced by hydroxyl radical but also by iron directly [18]. Moreover, the presence of organic solvent inhibits Fenton’s reaction and prevents DNA strand breaks. Therefore, questions still remain as to the actual effect of iron and ethylenediaminetetraacetic acid (EDTA) in DNA degradation, indeed Engelmann *et al.* have clearly shown that iron, EDTA and H_2_O_2_ ratio modify the Fenton reaction output [19]. The objective of this study was to establish the optimal conditions for the plasmid nick assay related to concentration of Fenton reagents and incubation time for various solvents used for the extraction of polyphenolics. With this established conditions the antioxidant and prooxidant effects of water, methanolic and acetonic extracts of *Oenocarpus bataua* (Ob) an Amazonian palm berry [20] and leaves of green tea (*Camelia sinensis* (Cs)) [8,21] were evaluated and compared to classical antioxidant assays.

## 2. Results and Discussion

### 2.1. In Vitro Chemical Antioxidant Capacity of Oenocarpus bataua and Camellia sinensis

To evaluate the antioxidant properties of *Oenocarpus bataua* and *Camellia sinensis*, extracts were assessed through chemical assays, FRAP, ORAC and DPPH assays to find out which mechanisms were involved. Moreover, as polyphenols are often suspected to be responsible for antioxidant capacity, the total phenolics content (TPC) were also determined. All results are presented in Table 1. The DPPH, ORAC and FRAP assays revealed a better antioxidant capacity of aqueous, methanolic, and acetonic Cs extracts compared to corresponding Ob extracts (Table 1). In a different way, the TPC measurement showed a greater polyphenolics contents for aqueous and acetonic Cs extracts compared to Ob extracts and similar total polyphenolics contents between Cs and Ob methanolic extracts (Table 1). To measure the extract capacities to protect a biological matrix such as DNA from oxidative stress and compare them to chemical tests, we decided to use the DNA nicking assay for non-site-specific •OH scavenging activity. For this assay, the parameters that need to be optimized were iron/EDTA ratio and the solvent percentage.

**Table 1:** *Oenocarpus bataua* and *Camellia sinensis* total phenolics content and *in vitro* antioxidant capacity.

Assay	TPC	DPPH	ORAC	FRAP
Biological relevance	Reduction of phosphomolybdique and phosphotungstique complexes	DPPH radical scavenging activity	ROO scavenging activity (radical chain breaking)	Fe^3+^ reduction (Fe^2+^ production)
Extract	(µg GAEq/mg DE)	(µmol TEq/g DE)	(µmol TEq/g DE)	(mmol Fe(II)Eq/g DE)
Cs (W)	364.6 ± 31.8	2741 ± 191	4941 ± 167	7.0 ± 0.3
Ob (W)	107.6 ± 6.2	424 ± 3	2189 ± 163	1.8 ± 0.1
Cs (M)	275.5 ± 22.5	2927 ± 193	6628 ± 86	7.5 ± 0.4
Ob (M)	306.5 ± 26	2054 ± 100	3708 ± 359	4.8 ± 0.1
Cs (A)	371.3 ± 11.4	3486 ± 191	6375 ± 107	7.9 ± 0.6
Ob (A)	183.9 ± 7.2	1325 ± 99	2132 ± 104	2.7 ± 0.2

TPC: Total Phenolics Content; GA: Gallic Acid; T: Trolox; Eq: Equivalent; DE: Dry Extract; W: water extract; M: methanol/water extract (70/30, *v*/*v*); A: acetone/water extract (70/30, *v*/*v*); error represent ± SD (*n* = 3).

### 2.2. Water pUC18 DNA Nicking Assay

**Figure 1:**
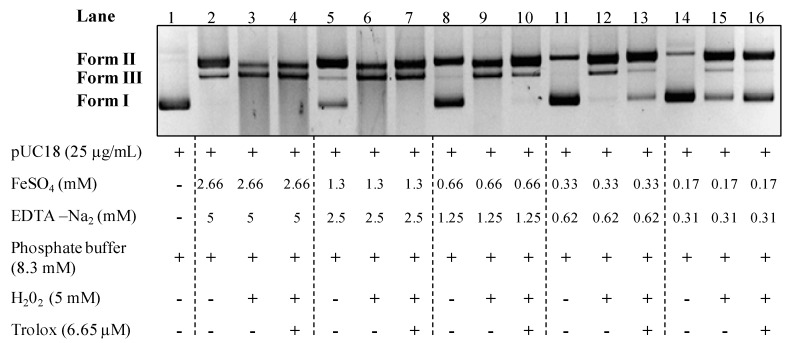
Aqueous DNA nicking assay optimization. Form I—supercoiled double stranded DNA; Form II—open circular DNA; Form III—nicked linear DNA. All the reaction mixtures were incubated for 20 min at 37 °C.

In their study dealing with iron effects on DNA strand breaks, Flemmig and Arnhold show that plasmid strand breaks is not mediated by hydroxyl radical produced from hydrogen peroxide in Fenton reaction but essentially by iron [18]. To determine working conditions so that DNA degradation is induced by •OH, in our study the reaction mixture proposed by Kitts *et al.* was used [17]. In order to observe pUC18 strand breaks only induced by •OH production, an assay with no iron effect was developed (Figure 1). To that end, the mixture were incubated 20 min at 37 °C and various EDTA and FeSO_4_ concentrations were tested keeping a molar ratio of 0.53 between EDTA and FeSO_4_ as specified by Repine *et al.* [22]. In this assay, we looked for a condition wherein the supercoiled form (form I) was totally degraded by H_2_O_2_ addition and then protected by adding an antioxidant compound, the trolox. In this approach, final concentrations of 0.33 mM of FeSO_4_ and 0.62 mM of EDTA were identified as optimal conditions to evaluate aqueous extracts •OH nicking protection capacity (lanes 11–13) (Figure 1). However, using this conditions we can notice that using FeSO_4_ and EDTA alone induce the formation of plasmid open circular form (form II), but did not induce plasmid linearization (form III) (lane 11) (Figure 1). These results led us to quantify the percentage of pUC18 form I (native form) and form III (nicked linear DNA) to evaluate both antioxidant or prooxidant capacity of extracts. Due to the slight over or under formation of the form II we chose to do not quantify its formation. The results obtained with pUC18 plasmid in this study confirmed the iron effect on strand breaks of the pBR322 plasmid already shown in Flemmig and Arnhold’s work [18]. Actually, if the iron concentration is not adapted, both plasmids (pUC18 and pBR322) are mostly cleaved by iron instead of •OH induced by the Fenton reaction.

### 2.3. Evaluation of Antioxidant and Prooxidant Capacity of Trolox, Quercetin and Gallic Acid

**Figure 2:**
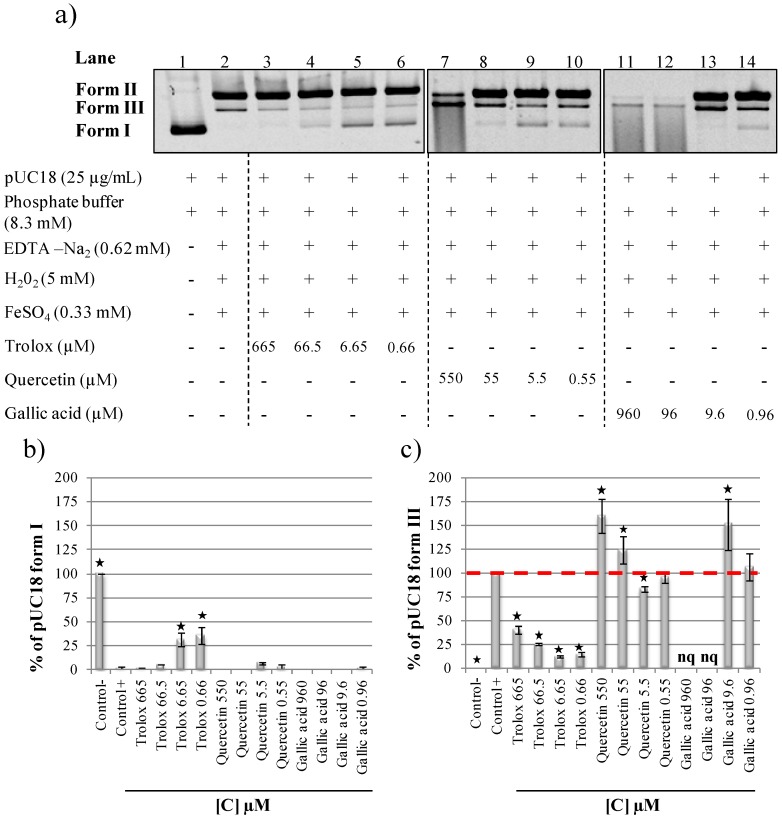
(**a**) Strand breaks protective capacity of aqueous trolox, quercetin and gallic acid samples; (**b**) Quantification of pUC18 form I protection; (**c**) Quantification of pUC18 form III formation. All the reaction mixtures were incubated 20 min at 37 °C. Asterisks indicate significant differences between control+ and the other assays at *p* < 0.05. nq: not quantifiable. Form I—supercoiled double stranded DNA; Form II—open circular DNA; Form III—nicked linear DNA.

The radical scavenging capacities of three pure compounds well known for their *in vitro* or *in cellulo* antioxidant or prooxidant activities were tested to improve our assay condition and identify good antioxidant references (Figure 2a). Trolox, a water-soluble analog of vitamin E, and two phenolic compounds were selected, all standards were dissolved in water. Trolox protected pUC18 form I from hydroxyl radical degradation at 6.65 and 0.66 µM (lanes 5 and 6). Quercetin presented dual behavior with a potent antioxidant activity at 5.5 and 0.55 µM (lanes 9 and 10), but a prooxidant activity at higher concentrations of 550 and 55.5 µM (lanes 7 and 8). Gallic acid presented prooxidant activity at 960, 96 and 9.6 µM (lanes 11–13). In order to measure the radical scavenging effect of these three compounds on pUC18 strand breaks, pUC18 form I and form III percentages on gel were quantified (Figure 2b,c). Low trolox and quercetin concentrations (6.65 and 5.5 µM) reduced pUC18 native form I degradation (with respectively 25% and 5% of form I protection compared to the positive control) (Figure 2b). Using form III quantification, trolox was found to be antioxidant in a dose dependant manner at all concentrations tested (Figure 2c), whereas quercetin was found to be prooxidant at 550 and 55 µM and antioxidant at 5.5 µM (Figure 2c). pUC18 degradation with gallic acid at 960 and 96 µM (form I and form II total disappearance) clearly showed prooxidant activities but this high degradation prevented a significant quantification of gallic acid form III formation (Figure 2a,c). However, gallic acid presented a quantifiable prooxidant activity at 9.6 µM (Figure 2c). The expected dual antioxidant or prooxidant behaviors of quercetin and gallic acid were observed in the present DNA nicking assay and validate our conditions. For example, Girard-Lalancette *et al.* showed that trolox, quercetin and gallic acid present in a cellular assay a dose dependant antioxidant activity [23]. In their assay they also found that trolox were antioxidant between 0.06 and 62.5 µM and prooxidant between 250 and 1000 µM. Gallic acid was found to be antioxidant between 0.2 and 1 µM and quercetin between 0.004 and 0.06 µM. The range of antioxidant capacity of gallic acid trolox and quercetin in cellular assay is comparable to our results with the DNA assay. In the literature, the prooxidant capacity of quercetin, gallic acid and other polyphenols is supposed to be directly linked to their ability to bind and reduce Fe^3+^ to regenerate Fe^2+^ which will then produce more hydroxyl radicals [24,25,26]. Due to trolox’ protective effect, at all concentrations tested it was chosen as strand breaks protection control for the next DNA nicking assays.

### 2.4. Organic Solvents Effect on pUC18 DNA Nicking Assay

**Figure 3:**
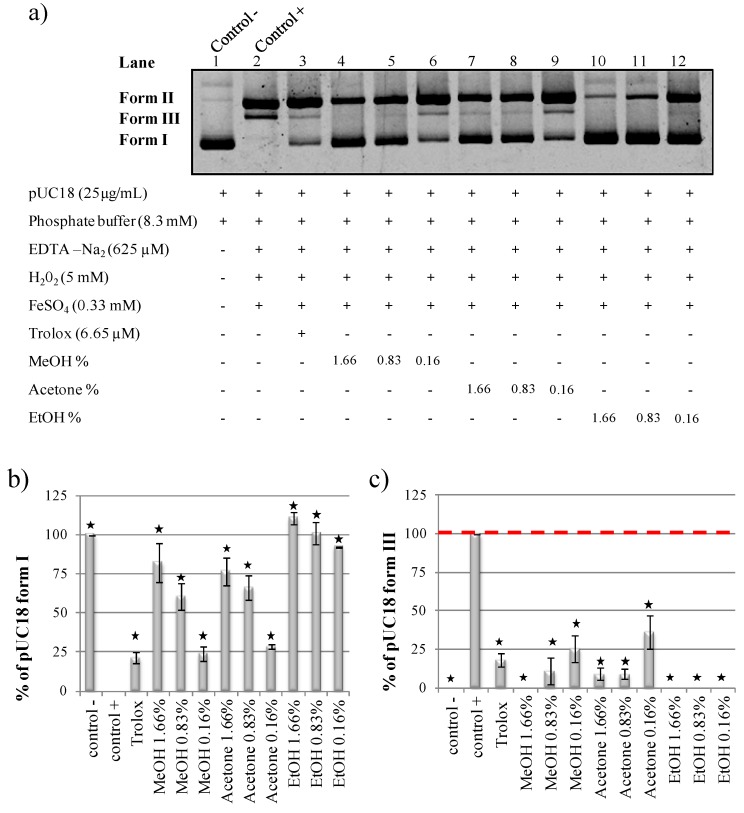
(**a**) Strand breaks protective capacity of methanol, acetone and ethanol; (**b**) Quantification of pUC18 form I protection; (**c**) Quantification of pUC18 form III formation. All the reaction mixtures were incubated for 20 min at 37 °C. Asterisks indicate significant differences between control+ and the other assays at *p* < 0.05. Form I—supercoiled double stranded DNA; Form II—open circular DNA; Form III—nicked linear DNA.

Good extraction yields for antioxidant molecules like polyphenols are often obtained with organic solvents like acetone, methanol or ethanol [27,28]. Unfortunately, organic solvents are also well known to have a DNA protective effect in this kind of assay (Figure 3a). Because the DNA backbone is negatively charged metal ions as Fe^2+^ can bind DNA and can catalyze the Fenton reaction although iron binding to DNA can also cause DNA damage. Organic solvents may impair this interaction thereby preventing the Fenton reaction from occurring. We investigated whether it was possible to quantify antioxidant activities of methanolic, acetonic, ethanolic or DMSO solubilized extracts in our pUC18 DNA nicking assay. To do so, in a first step, various final percentages of solvent (1.66%, 0.83% and 0.16%) were added in the DNA nicking assay mixture to evaluate their effects (Figure 3a). As described in the literature, methanol, acetone and ethanol were strong antioxidants, even at 0.16% (Figure 3a). Methanol is known to inhibit DNA degradation by reacting with hydroxyl radicals and being oxidized into formaldehyde and formic acid [29]. Ethanol is also known to be a good hydroxyl radical scavenger [30]. The DMSO antioxidant capacity was also tested and the same antioxidant profile as ethanol was observed (data not shown). This result was in accordance with a previous work demonstrating that DMSO prevents total DNA nicking, mediated by iron/hydrogen peroxide-generated hydroxyl radical [22]. Quantification of methanol, acetone and ethanol pUC18 form I protection in Figure 3b showed a dose dependent effect of solvent percentage on antioxidant capacity. Quantification of pUC18 form III formation confirmed the methanol, acetone and ethanol having high antioxidant activity (Figure 3c). The antioxidant property of organic solvents is linked to their ability to react with free radicals, preventing them from reacting with DNA and causing damages. Because DMSO and ethanol were DNA protective at even 0.16% (Figure 3b,c). Therefore, in a second step we looked only at improving reaction conditions for acetone and methanol. The objective was to determine conditions with no solvent effect.

### 2.5. Methanol and Acetone pUC18 Strand Breaks Assay

**Figure 4:**
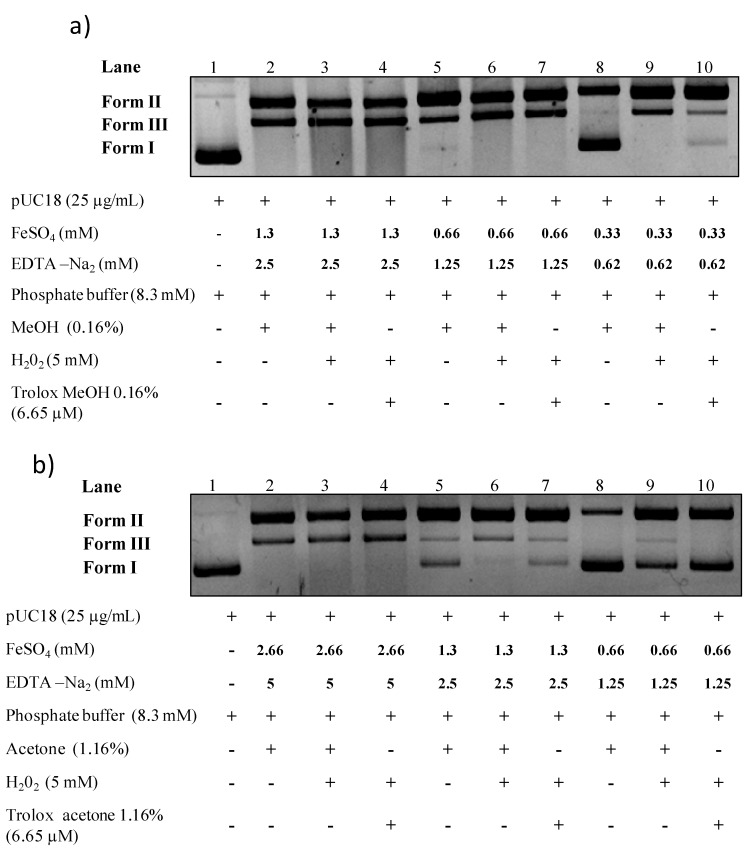
(**a**) Development of a pUC18 DNA nicking assay with methanolic solvent; **(b**) Development of a pUC18 DNA nicking assay with acetonic solvent. Reaction mixtures were incubated for 15 min at 37 °C for acetone and for 1.5 h for methanol. Form I—supercoiled double stranded DNA; Form II—open circular DNA; Form III—nicked linear DNA.

In order to test methanol and acetone extracts’ antioxidant capacities, the method described in Figure 1 was applied and various solvent percentages, reaction times and iron/EDTA concentrations were tested (data not shown). During these assays, to remove solvent effects, cautions were taken to perform control with exactly the same amount of methanol or acetone in all the tested lanes (Figure 4).

The optimized conditions for methanol extracts were: 0.16% solvent, incubated for 1.5 h at 37 °C with 0.33 mM of FeSO_4_ and 0.62 mM of EDTA (Figure 4a). The optimized conditions for acetone extracts, were 1.16% solvent, incubated for 15 min at 37 °C and 1.3 mM of FeSO_4_ and 2.5 mM of EDTA (Figure 4b). Higher acetone concentrations were then tolerated. These results were surprising since the protective effect of methanol and acetone against form I were similar (Figure 3a) and should induce the same behavior in the DNA nicking assay. These differences could come from the OH group present in the methanol which is supposed to confer a higher antioxidant capacity [31]. The optimized conditions identified for acetonic and methanolic extract were then used to evaluate the plant extracts protective capacity.

### 2.6. DNA Antioxidant and Prooxidant Capacity of Camellia sinensis and Oenocarpus bataua

**Figure 5:**
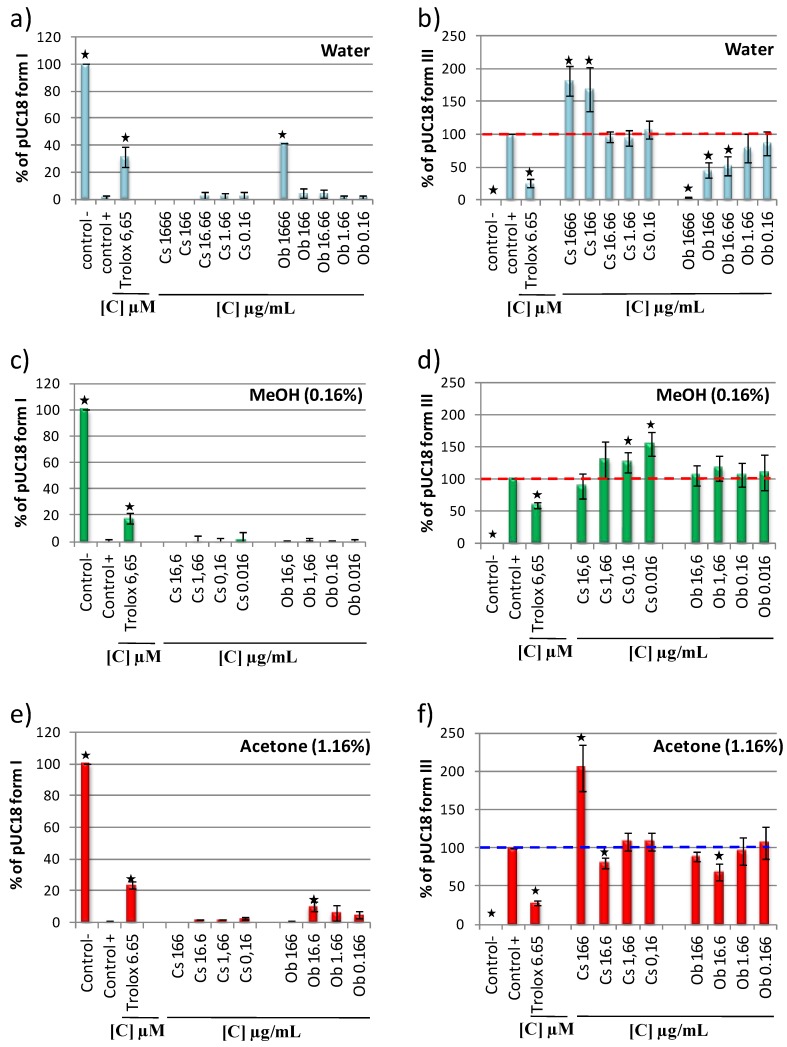
Quantification of pUC18 aqueous *Camelia sinensis* (Cs) and *Oenocarpus bataua* (Ob) extract (**a**) form I protection and (**b**) form III formation; Quantification of pUC18 methanolic *Camelia sinensis* (Cs) and *Oenocarpus bataua* (Ob) extract (**c**) form I protection and (**d**) form III formation; Quantification of pUC18 acetonic *Camelia sinensis* (Cs) and *Oenocarpus bataua* (Ob) extract (**e**) form I protection and (**f**) form III formation. Asterisks indicate significant differences between control+ and the other assays at *p* < 0.05. Data are representative of three assays, error bars represent SD.

DNA antioxidant or prooxidant capacities of Cs and Ob aqueous, methanol and acetone extracts were studied using the assay conditions identified in this study (Figure 1 and Figure 4). The pUC18 form I and form III were quantified on gel (gel not shown) and the relative content of form I and III were compared between the extracts (Figure 5). A dose dependent assay was performed with successive dilutions of Cs and Ob extracts. The pUC18 plasmid incubated with Fenton reagent was used as a positive control, the plasmid incubated without the Fenton reagent as a negative control and the plasmid incubated with the Fenton reagent and trolox was used as a strand breaks protection control. With aqueous extracts, no form I protection was observed for Cs extract (Figure 5a). Quantification of form III formation showed for a concentration of 1666 and 166 µg/mL, that Cs extract was prooxidant while at the other tested concentrations, Cs extracts were neither antioxidant nor prooxidant (Figure 5b). For a concentration of 1666 mg/mL (Figure 5a), 166 and 16.6 µg/mL (Figure 5b) Ob aqueous extracts were antioxidant. As crude methanolic and acetonic extracts were dissolved in 70/30 *v*/*v* methanol water or acetone water solution to conserve the appropriate solvent percentage in the assay, the higher concentrations tested were 16.6 µg/mL for methanolic extracts and 166 µg/mL for acetonic extracts. With methanol extracts, no significant antioxidant capacity was quantified for Cs and Ob (Figure 5c,d) but a prooxidant activity of Cs extracts at 0.16 and 0.016 µg/mL was quantified (Figure 5d). Experiments with acetone extracts showed that Cs was slightly antioxidant at 16.6 µg/mL and prooxidant at 166 µg/mL (Figure 5e,f), while Ob was antioxidant at 16.6 µg/mL (Figure 5e,f). In these condition assays, quantification of pro or antioxidant capacity of Cs and Ob extracts (water, methanol and acetone) revealed that Cs was mostly prooxidant whereas Ob was principally antioxidant (Figure 5). An explanation for pro or antioxidant capacity of Cs and Ob could be their composition. Actually, in Ob stilbenes (3080 µg/g of dry weight) anthocyanes (470 µg/g of dry weight) and procyanidins (18,000 µg/g of dry weight) are identified [20], whereas in green tea (−)-Epigallocatechin-3-gallate (EGCG) (129,000 µg/g of dry weight) and (−)-Epicatechin-3-gallate (ECG) (46,000 µg/g of dry weight) are found [32], these latter two compounds have been identified to induce *in vitro* DNA strand scission when they are complexed with Fe^3+^ [25,26]. Interestingly, at the same concentrations, tested Cs or Ob aqueous methanolic and acetonic extract showed different antioxidant capacities. These differences are not surprising since extraction solvents are well known to play a direct role on extract composition (yield and nature of extracted molecules). In this study, no obvious links were found between an antioxidant (or prooxidant) capacity in DNA nicking assay and TPC, DPPH and ORAC assay. However, a potential link could be suspected between the FRAP capacity and the protective capacity in the DNA assay. Actually, Table 1 shows that extracts with the lower FRAP capacity (Ob (W) and Ob (A)) were the most protective in the DNA assay and extracts with the higher FRAP capacity (Cs (W) and Cs (A)) the less protective (Figure 5). This behavior may come from the fact that when an extract had a good capacity to reduce Fe^3+^ in Fe^2+^, this extract will induce much more hydroxyl radicals which will lead to a greater DNA degradation. These results reinforce the idea that, to evaluate antioxidant properties of natural extracts various *in vitro* as *in vivo* assays should be performed.

## 3. Experimental Section

### 3.1. Plant Material

Rip fruits from Amazonian palm tree *Oenocarpus bataua* were harvested during January 2013 in the city of Macouria (2 km away from Cayenne, French Guiana). They were ready for consumption, free of physical damages, injuries from insects and fungal infections. After peeling, *Oenocarpus bataua* pulps were Freeze-dreied and crushed using a Thermomix TM 31 (Vorwerk, France). The obtained dried matter was then stored at −20 °C to limit degradation until analysis. For *Camellia sinensis*, commercial biological farming dried powder was purchased from supermarket.

### 3.2. Extracts Preparation

Freeze-dried powders of *Oenocarpus bataua* and *Camellia sinensis* (2.5 g) were extracted four times in 50 mL of water (H_2_O) solutions, acetone/water (Ac/H_2_O) solutions (70/30, *v*/*v*) or methanol/water (MeOH/H_2_O) solutions (70/30, *v*/*v*) under sonication (130 kHz, 10 min) at room temperature. Then, each extract was centrifuged (5000× *g*, 10 min, 4 °C) and supernatants were combined after filtration. Organic solvents were evaporated in a rotary evaporator using a bath at 40 °C, aqueous extracts were lyophilized. Finally, dried extracts were weighed for yield and then dissolved in their extraction solvent at a concentration of 40 mg/mL.

### 3.3. Chemicals

Analytical grade quality methanol, acetone and ethanol were used (Carlo Erba, France), fluorescein disodium salt, 2,4,6-tris(2-pyridyl)-s-triazine (TPTZ) and glacial acetic acid were obtained from Fluka Sigma–Aldrich (Steinheim, Germany). 2,2-Diphenyl-l-picrylhydrazyl (DPPH), 6-hydroxy-2,5,7,8-tetramethylchromane-2-carboxylic (Trolox), 2,2'-azobis(2-amidino-propane), dihydrochloride (AAPH) and quercetin came from Acros Organics (Geel, Belgium). K_2_HPO_4_, KH_2_PO_4_ and FeCl_3_ came from Fisher Scientific (Villebon sur Yvette, France). Gallic acid and iron (II) heptahydrate sulfate were purchased from Alfa Aesar (Ward Hill, MA, USA). Folin–Ciocalteu reagent, dimethyl sulfoxide (DMSO), Na_2_CO_3_, ethylenediaminetetraacetic acid disodium salt dihydrate (EDTA-Na_2_) and hydrochloric acid came from CARLO ERBA reagents (Reuil, France). Hydrogen peroxide and sodium acetate trihydrate came from Chimix (Chassieu, France).

### 3.4. Optimization of DNA •OH Nicking Assay Conditions of Aqueous and Organic Extracts

To identify condition assay in DNA protective capacities of water, acetone/water and methanol/water extracts, 4 μL of pUC18 plasmid DNA (150 μg/µL) was mixed with 4 µL each of phosphate buffer (H_2_PO_4_, 50 mM, pH 7.4), H_2_O_2_ (30 mM) and variable concentrations of FeSO_4_ and EDTA-Na_2_. 4 µL of trolox solution at 0.01 mg/mL was used as DNA protection control for water assay. For acetone/water and methanol/water assay, 4 µL of trolox solution at 0.01 mg/mL with respectively 7% and 1% of acetone and methanol were used as protection control. For this assays, cautions were taken to maintain the same solvent percentage in each mixture. Final volume of each reaction mixture was brought to 24 μL by water addition in a 500 μL microcentrifuge tube. Reaction mixtures were respectively incubated for 20 min at 37 °C for water condition, or for 15 min at 37 °C for acetone/water condition and for 1.5 h at 37 °C for methanol/water condition. Following incubation, 2 μL of loading dye was added to the incubated mixture, and 10 μL was loaded onto a 1% (*w*/*v*) agarose gel. Electrophoresis was conducted at 100 volts in Tris-Acetate-EDTA•Na_2_ (TAE) buffer (0.04 M tris-acetate and 1 mM EDTA, pH 7.4) using a DNA subcell (Bio-Rad). The agarose gel was stained with ethidium bromide for 15 min. DNA bands were visualized with a Bio-Rad Gel Doc™ XR.

### 3.5. DNA Nicking Assays of Pure Compounds and Plant Extracts

To assess antioxidant capacities of organic solvents (acetone, methanol, ethanol), pure compounds (trolox, gallic acid, quercetin) and aqueous extracts of *Oenocarpus bataua* and C*amellia sinensi*s, 4 μL of extracts at various concentrations were mixed with 4 µL each of pUC18 plasmid DNA (150 μg/µL), phosphate buffer (H_2_PO_4_, 50 mM, pH 7.4), H_2_O_2_ (30 mM), FeSO_4_ (2 mM) and EDTA-Na_2_ (3.75 mM). Water trolox solution at 0.01 mg/mL was used as protection control for *Oenocarpus bataua* and *Camellia sinensi*s assays. To assess *Oenocarpus bataua* and *Camellia sinensi*s acetone/water, antioxidant capacities, 4 μL of extracts with 7% acetone were mixed with 4 µL each of pUC18 plasmid DNA (150 μg/µL), phosphate buffer (H_2_PO_4_, 50 mM, pH 7.4), H_2_O_2_ (30 mM), FeSO_4_ (8 mM) and EDTA-Na_2_ (15 mM). 4 µL of trolox solution at 0.1 mg/mL with 7% acetone was used as protection control. To assess *Oenocarpus bataua* and *Camellia sinensi*s methanol/water antioxidant capacity 4 μL of extract with 1% methanol were mixed with 4 µL each of: pUC18 plasmid DNA (150 μg/µL), phosphate buffer (H_2_PO_4_, 50 mM, pH 7.4), H_2_O_2_ (30 mM), FeSO_4_ (2 mM) and EDTA-Na_2_ (3.75 mM). 4 µL of trolox solution at 0.1 mg/mL with 1% of methanol was used as protection control. All reaction mixtures were performed in a 500 μL microcentrifuge tube and precautions were taken to have the same solvent percentage in each mixture. Final volume of each reaction mixture was brought to 24 μL by addition of appropriate solvent and incubated respectively 20 min, 15 min and 1.5 h at 37 °C for aqueous, acetone and methanol extracts. After incubations, 2 μL of loading dye was added to the incubated mixture, and 10 μL were loaded onto a 1% (*w*/*v*) agarose gel. Electrophoresis was conducted at 100 volts in Tris-Acetate-EDTA•Na_2_ (TAE) buffer (0.04 M tris-acetate and 1 mM EDTA, pH 7.4) using a DNA subcell (Bio-Rad). The agarose gel was stained with ethidium bromide for 15 min. DNA bands were visualized and photographed with a Bio-Rad Gel Doc™ XR.

### 3.6. Quantification of pUC18 Form I and Form III Percentage 

In order to quantify extracts pro or antioxidant capacities, pUC18 form I and form III bands were pictured with the Bio-Rad Gel Doc™ XR and their intensity were quantified with the ImageJ software. Afterward the negative controls form I (supercoiled) intensity were used as 100% of form I protection and the positive controls form III (nicked linear) intensity were used as 100% of form III formation. The negative control was pUC18 incubated alone and the positive control pUC18 incubated with Fenton reagent.

### 3.7. Phytochemical Analysis

#### Total Phenolic Content (TPC)

The TPC procedure was operated according to Arnous *et al.* (2002) [33] with some modifications. Phenolic contents of crude water, acetone/water and methanol/water extracts were determined using Folin-Ciocalteu reagent and gallic acid as phenolic standard. In brief, water (2300 μL), 100 μL of appropriate dilutions of *Oenocarpus bataua* or *Camellia sinensis* extracts and 450 μL of Folin-Ciocalteu reagent were mixed and 150 μL of a 20% sodium carbonate (Na_2_CO_3_) solution was added at room temperature. After incubation for 2 h, the blue color that developed was recorded at 750 nm. The TPC were expressed in µg gallic acid equivalent (GAEq) per milligram of dried extract (µg GAEq/mg DE).

### 3.8. Evaluation of in Vitro Antioxidant Activity by Chemical Assays

#### 3.8.1. DPPH Assay

DPPH radical scavenging activity of the extracts were measured using the method from Brand-Williams *et al.* (1995) [12] with some modifications. Reduction of the DPPH radical by an extract or a standard was followed by the decrease of its absorbance at 520 nm. Five different dilutions of the extracts were prepared and an aliquot of 100 μL of diluted sample was added to 3900 μL DPPH solution (1 × 10^−4^ M) and vortexed. A DPPH methanolic solution was prepared as control. After 2 h of incubation in darkness and at room temperature, absorbance of control and samples was measured at 520 nm. Trolox was used as reference and the DPPH radical scavenging activity was expressed in μM Trolox equivalent/g of dried extract (TEq μmol/g DE).

#### 3.8.2. FRAP Assay

Total antioxidant activity was assayed with the original method of Benzie and Strain [13], while some modifications were also made. A FRAP solution was prepared by adding 25 mL of acetate buffer (pH 3.6 at 300 mM), 2.5 mL of acidic TPTZ solution (10 mmol/L in HCl at 40 mmol/L) and 2.5 mL of fresh FeCl_3_ (20 mmol/L in water). 3000 µL of this FRAP solution was mixed with 300 μL of distilled water and 100 μL of reference (Fe (II)) or test sample at different concentrations (*n* = 3). Spectrophotometric analyses were performed at 37 °C. The reaction was monitored for up to 30 min and absorbance was measured at 595 nm. Results were expressed in mmol Fe (II) equivalent/g of dried extract (mmol Fe (II) Eq/g DE).

#### 3.8.3. ORAC Assay

The ORAC procedure was done according to the method of Ou *et al.* (2001) [11] with some modifications. The assay was carried out on a Cary Eclipse Fluorescence Spectrophotometer (Varian, France) in phosphate buffer pH 7.4 (75 mM) at 37 °C. Fluorescein was used as fluorescent probe, AAPH as a peroxyl radical generator and Trolox as a standard at 20–120 µM. 200 µL of blank, standard or sample (diluted in 75 mM phosphate buffer) and 2 mL of fluorescein 30 nM were mixed and preincubated at 37 °C for 15 min. Then, 200 µL of 153 mM of AAPH were added. Fluorescence was measured every minute for 60 min at an emission wavelength of 520 ± 5 nm and an excitation wavelength of 485 ± 5 nm. All samples were analyzed at three different dilutions and run in duplicates with a relative standard deviation <10%. The quantification of the antioxidant activity was based on the calculation of the area under the curve deduced from that of the blank. Antioxidant activity was expressed as µmol of trolox equivalents/g of dried extract (µmol TEq/g DE).

### 3.9. Statistical Analysis

During the DNA assays, an average error of 15% in quantifications was found. All tests were conducted in triplicate. The results were expressed as means ± SD. For DNA assays, form I protection or form III formation were compared respectively to form I and form III positive controls using the two-tailed Mann–Whitney U test at *p* < 0.05 to indicate significant differences between positive control and extracts tested.

## 4. Conclusions

This study shows that *in vitro* DNA nicking assays should be performed with the appropriate controls (positive control, negative control and solvent effect control). To correctly measure the protective capacity of aqueous, methanolic and acetonic natural extracts, a method was proposed to remove both iron prooxidant activity and solvent antioxidant capacity playing on three factors: time reaction, solvent concentration and iron/EDTA concentration. Our data confirmed the work of Engelmann *et al.*, which presented the central role of iron, EDTA and H_2_O_2_ in the intensity of oxidative stress. We showed that not all the solvents could be used in this DNA nicking assay due to their high protective effect (DMSO and ethanol). The application of our DNA nicking method to *Ob* fruit and *Cs* leaves extracts (water, methanol and acetone) showed that *Cs* extracts were prooxidant whereas *Ob* extracts were antioxidant. Nonetheless, these results are not totally concordant with the classical chemical *in vitro* assay (ORAC, DPPH) performed in this study, which showed that *Cs* and *Ob* were both good antioxidant plants. However, the FRAP assay performed with *Cs* and *Ob* highlighted a possible link between plant extracts capacity to protect or not DNA and their capacity to reduce Fe^3+^ in Fe^2+^. In fact, in the FRAP assays, the extracts with the better FRAP value were mostly prooxidant and those with the worst value antioxidant. The DNA nicking assay is a powerful tool that can be used to measure the antioxidant and prooxidant effects of an extract on a biologically important and physiologically relevant cellular component such as DNA. To better understand the pro or antioxidant capacities of the extracts using the DNA nicking assay, the iron binding activity should be further investigated.
